# Cue-Recruitment for Extrinsic Signals after Training with Low Information Stimuli

**DOI:** 10.1371/journal.pone.0096383

**Published:** 2014-05-07

**Authors:** Anshul Jain, Stuart Fuller, Benjamin T. Backus

**Affiliations:** Graduate Center for Vision Research, State University of New York College of Optometry, New York, New York, United States of America; University of Akron, United States of America

## Abstract

Cue-recruitment occurs when a previously ineffective signal comes to affect the perceptual appearance of a target object, in a manner similar to the trusted cues with which the signal was put into correlation during training [1], [2]. Jain, Fuller and Backus [3] reported that *extrinsic* signals, those not carried by the target object itself, were not recruited even after extensive training. However, recent studies have shown that training using weakened trusted cues can facilitate recruitment of *intrinsic* signals [4]–[7]. The current study was designed to examine whether extrinsic signals can be recruited by putting them in correlation with weakened trusted cues. Specifically, we tested whether an extrinsic visual signal, the rotary motion direction of an annulus of random dots, and an extrinsic auditory signal, direction of an auditory pitch glide, can be recruited as cues for the rotation direction of a Necker cube. We found learning, albeit weak, for visual but not for auditory signals. These results extend the generality of the cue-recruitment phenomenon to an extrinsic signal and provide further evidence that the visual system learns to use new signals most quickly when other, long-trusted cues are unavailable or unreliable.

## Introduction

The visual system is adaptable. Examples of this ability include improvement in performance over time [8], [9], negative adaptation aftereffects (e.g. tilt aftereffect [10]) and other modulations of perceptual biases [11]–[14]. Another important form of adaptation is learning to use a sensory signal in a new way, as a cue for constructing appearance. This phenomenon has been called cue-recruitment [1], [2], [6]. Since 2006 it has been demonstrated that many signals can be recruited as cues using an associative learning paradigm, including location [2], [4], [6], [11], [15]–[17], translation direction [2], surface-texture [6], vertical disparity [7], color of illumination [18], object shape [5] and motor actions [19].

In a common version of the cue recruitment experimental paradigm, participants are trained using trials that contain otherwise ambiguous stimuli, that are disambiguated using long-trusted cues. Critically, the new signal to be learned as a cue is put into statistical correlation with the long-trusted cues during the training, which can result in the new signal acquiring the ability to disambiguate appearance in a manner similar to the long-trusted cues.

Some signals, such as retinal location or object translation direction, typically show greater learning than other signals such as object shape or vertical disparity, as measured by their ability to bias the perceived rotation direction of an ambiguously rotating 3D object. The strength of learning of the location signal itself has also been shown to vary across experiments with different trained perceptual consequences. For example, the location signal biased the perceived 3D rotation direction of a Necker cube much more strongly than the perceived configuration of stationary 3D shape [6] or 3D interpretation of self-generated optic flow [11].

It is unlikely that the strength of learning varied across these studies due to uncontrolled stimulus differences across experiments. The differences in learning due to controlled factors, such as the new signal to be learned, whether the session started with ambiguous trials [4], [17], [20], or whether the object was moving, were large; whereas effects are robust to changes in other factors, such as overall size of the display [4], [17], [20]. Factors that are intrinsic to the perceptual system play a role. Thus, Jain and Backus [6] argued that lack of motion was the factor that reduced the strength of learning when location was recruited as cue for stationary 3D shapes.

It has long been known that animals learn some associations more readily than others. In their classic study, Garcia and Koelling [21] showed that rats learned to associate illness with tastes more readily than with auditory or visual signals. On the other hand, rats learned to associate pain more readily with auditory or visual signals than with tastes. Garcia and Koelling argued that the system has prior belief about which signals are predictive about different states of the world; the learning rate is higher for plausible predictors of a given state than for implausible ones.

Similarly, Jain et al. [3] found in human perception that extrinsic signals, i.e. signals that are not carried by nor visually connected to the object whose appearance was being trained, were not recruited as cues. These unlearned signals included the rotation direction of an annulus of dots, in the plane of the display, that surrounded the ambiguously rotating cube; the location of a luminous disc, relative to the cube; and auditory signals.

In these cue-recruitment studies, the learning was under the control of the perceptual system, since all signals were supra-threshold [2]. Thus, whether learning occurs can be interpreted as showing whether the system implicitly believes the signal is likely, a priori, to be informative about the estimated property of the environment [1], [2], [22]. Nevertheless, learning must be done by specific neural mechanisms, and recent studies on cue-recruitment show that specifics of the training protocol can affect whether learning occurs. For example, the use of low information (i.e. weakly disambiguated) training stimuli can cause stronger learning [4], [17], [20], and can also enable recruitment of signals such as surface texture [6], vertical disparity [7] and shape [5] that were not recruited when training stimuli were strongly disambiguated. These previous studies employed intrinsic signals, i.e. signals carried by the same display elements as the object itself [3]. The current study was designed to test whether training with low information stimuli can cause extrinsic signals to be recruited as cues to visual appearance. We considered two extrinsic signals, one visual (unimodal) and one auditory (crossmodal). Specifically, we tested whether an annulus of dots rotating in the plane of the display, or an auditory pitch glide, can be recruited as a cue to bias the perceived rotation direction of a Necker cube rotating about the vertical axis.

## Materials and Methods

### Participants

Forty trainees participated in the experiments, fourteen in Experiment 1, six in Experiment 2A and twenty in Experiment 2B. All participants were naïve to the purpose of the experiments. All participants had normal or corrected-to-normal vision and normal hearing (self reported). We assessed each participant's stereoacuity using the TNO Stereo-acuity test to confirm that stereo-disambiguated training cubes would be seen as specified by disparity; all participants had a stereo acuity of 120 seconds of arc or less.

### Ethics Statement

The experiments were conducted in compliance with the standards set by the Institutional Review Board at SUNY College of Optometry. Participants gave their written informed consent prior to their inclusion in the study and were paid for their participation. All experimental procedures were approved by the Institutional Review Board at SUNY College of Optometry.

### Apparatus

All experiments were implemented on a Dell Precision 3400 computer (Windows platform) using the Python-based virtual reality toolkit Vizard 3.0 (WorldViz LLC, Santa Barbara, CA, USA). Visual stimuli were rear-projected onto a screen using either an Infocus LP350 projector (visual cue recruitment experiment, Experiment 1) or a Cristie Mirage S+ 4K projector (auditory cue recruitment experiment, Experiment 2A and 2B). Auditory stimuli were presented on a Bose® 161 speaker system driven by an AudioSource Stereo Amplifier AMP-100. The speakers were placed on either side of the screen along the horizontal midline. Participants were seated at a distance of 1.3 m from the screen for Experiment 1 and at a distance of 2.0 m from the screen for Experiment 2A and 2B. The visual and auditory cue-recruitment experiments were conducted in different rooms. In Experiment 1 we used an EyeLink I eye tracker (Missisauga, Ontario, Canada) to monitor fixation and record eye position.

### Stimuli and Procedure

#### Experiment 1 – Visual Cue-recruitment

**Figure 1:**
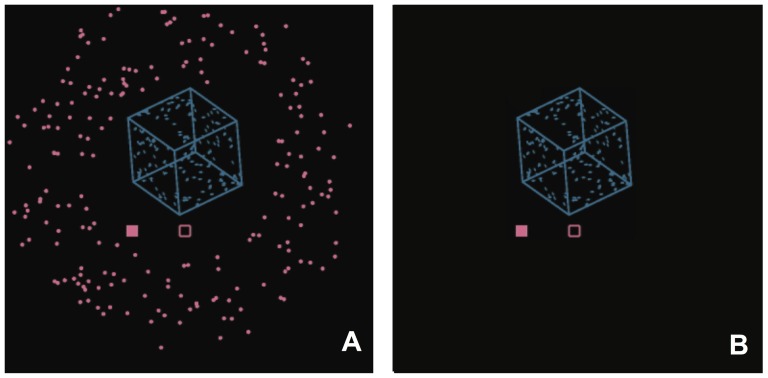
Design of stimuli used in the study. A) Layout of the stimulus used in Experiment 1. B) Layout of the visual stimulus used in Experiment 2.

The visual stimulus consisted of an orthographically projected (i.e. no perspective) wireframe (Necker) cube rotating about the vertical axis (Figure 1A). Each edge of the cube was a solid parallelepiped with a thickness of 0.6 cm and a length of 15 cm. The cube therefore subtended 12.4 degrees of visual angle. In order to stabilize perception of the cube as a single rigid object, each face of the cube was covered with 25 randomly placed dots. The cube was oriented such that one of the major diagonals was perpendicular to the axis of rotation. The cube was presented in two initial configurations, as “seen-from-above” or as “seen-from-below”. To satisfy these criteria, the yaw, pitch and roll were set to 50, 25 and 25 degrees respectively at the onset for the “seen-from-above” configuration and at 50, −25 and −25 degrees for “seen-from-below” configuration. These two configurations were balanced for each participant to avoid correlating them with cube rotation. The cube's angular velocity about the vertical axis was 72 degrees/second.

Left and right eye image segregation, which was necessary to display binocular disparities, was implemented using red-green anaglyphs and matching filter glasses. On training trials, the rotation direction was weakly disambiguated by using transient disparity signals. Disparity had the correct magnitude for the simulated cube, assuming a 6.2 cm interpupillary distance, but was presented only for 150 ms at the beginning of a training trial. After that, the left eye's image was extinguished and there were no disambiguating signals. Under these conditions, the apparent rotation direction established by the transient disparity generally perseverated to the end of the trial. On test trials, only one of the anaglyph images of the cube was presented (to the right eye).

A fixation square (2 cm×2 cm) was presented at the center of the screen and the cube was centered 15 cm (7.1 degrees) above the fixation square. The cube's center was simulated to be in the plane of the screen. All stimuli were presented as bright objects on a dark background. Concurrent with the cube stimulus on each trial, we presented a single probe dot (1 cm×1 cm) that translated horizontally in the screen plane through the fixation point, either leftwards or rightwards across a visual angle slightly larger than that of the cube. The dot traveled at approximately the same speed as the closest (or farthest) corner of the cube.

The new signal to be trained was a field of randomly placed dots in an annulus surrounding the cube. The annulus rotated within the plane of the display screen at the same angular speed as the cube rotated about its vertical axis, and its rotation direction was perfectly correlated with the cube's rotation direction on training trials. The polarity of the correlation was counterbalanced across participants. The dots had a mean lifetime of 100 ms. The field of dots and the cube were presented simultaneously after the participant confirmed that they had proper fixation (see Video S1).

#### General Procedure

The experiment consisted of two kinds of trials, Training trials and Test trials. On training trials, the perceived rotation direction of the cube was controlled using a brief pulse of disparity signal as described above to establish the correlation between the cube's rotation direction and the new signal (the rotation direction of the annulus of dots). On test trials the cube was ambiguous (no disparity) and was presented with one or the other value of the new signal. A trial consisted of the presentation of the cube stimulus, the probe dot, and the new signal, and lasted for 1.5 s.

The participants' task was to report whether the translation direction (leftward or rightward) of the probe dot was same as the front (closer to the participant) or back (farther away from the participant) side of the cube. Participants pressed the ‘2’ key to report that the front of the cube moved in the same direction as the probe dot and pressed the ‘8’ key to report that the back of the cube moved in the same direction as the probe dot. Because the dot's direction was randomly chosen on each trial, participant responses were decoupled from both perceived rotation direction and the new signal's values. This task was chosen to discourage participants from using complicated cognitive strategies in choosing their response. Post-experiment interviews confirmed that responses were mediated by the apparent rotation of the cube: none of the participants reported having noticed the correlation of training signal and rotation direction, much less having used it to respond. The next trial began 1 s after response. Participants were instructed to fixate on the fixation square throughout the experiment. We monitored fixation during the experiment. The eye-tracker was recalibrated before each of the five blocks.

**Figure 2:**
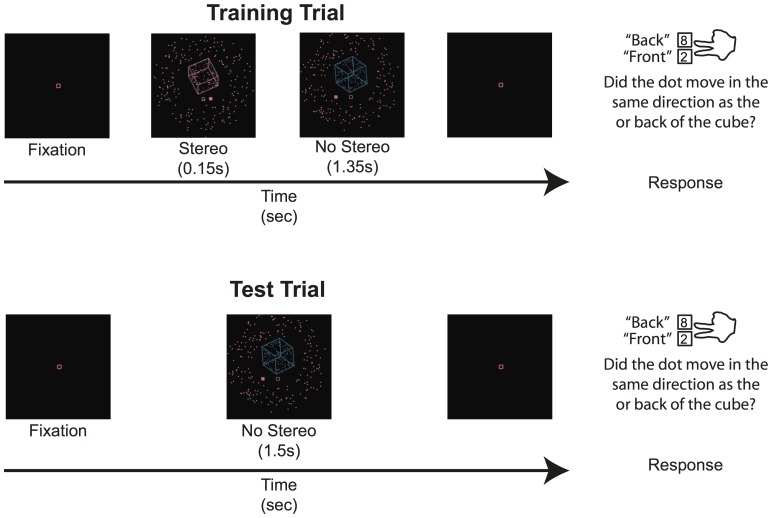
Structure of typical training and test trials presented in Experiment 1.

The experiment was conducted over a single session consisting of 480 trials split into five blocks of 96 trials each. The first block contained only training trials, to establish a perceptual history reflecting the correlation of the new signal and cube rotation before beginning to test with ambiguous cubes. The training trials for rightward and leftward rotations were presented equally often but pseudo-randomly sequenced. The sequence was constrained such that participants could not be presented with cubes rotating in the same direction on more than eight consecutive trials. The remaining blocks contained an equal mix of test and training trials presented pseudo-randomly. The sequence was constrained such that participants could never be presented with the same type of trial (test or training) on more than four consecutive trials. Figure 2 shows the structure of typical training and test trials presented in Experiment 1.

The 14 participants in Experiment 1 were randomly assigned to two groups: one for which clockwise rotation of the annulus specified leftward rotation of the cube, and one for which it specified rightward rotation of the cube, as a precaution to control for preexisting biases in perception within the population. In fact, both groups showed similar learning, so we did not find evidence of a preexisting bias to use annulus rotation direction as a cue for a particular cube rotation direction (see Results and Discussion). Similarly, in Experiment 2A, the 6 participants were randomly assigned to two groups: one for which upward pitch glide specified leftward rotation of the cube and one for which upward pitch glide specified rightward rotation of the cube.

#### Experiment 2 – Auditory Cue-recruitment

The visual stimuli in Experiments 2A and 2B were the same as in Experiment 1 after adjusting the size to account for the larger viewing distance, except that they rotated about the vertical axis at 120 degrees/second and did not contain the annulus of dots (Figure 1B).

In both Experiment 2A and 2B, the new signal to be trained was an auditory composite pitch glide presented simultaneously with visual onset of the stimulus. The pitch glide was formed with five harmonics. For an upward glide (Audio S1) frequencies [65.4 130.8 261.6 523.2 1046.4] Hz were increased linearly as a function of time up to [185 370 740 1480 2960] Hz, respectively, over the course of 1.5 s. The direction was reversed for a downward glide (Audio S2). The auditory stimulus amplitude was ramped up and down over 20 ms at the start and end of the stimulus, respectively, to avoid audible “clicks” caused by sudden onset and offset. The intensity of the auditory stimulus was set to supra-threshold but comfortable levels (the exact intensity is not critical to the aim of the study).

The procedure for Experiment 2A was the same as Experiment 1. The rotation direction on training trials was disambiguated using transient disparity signals presented at the beginning of the trial for 150 ms, just as in Experiment 1. The experiment was conducted over a single session consisting of 480 trials split into five blocks of 96 trials each with the first block containing only training trials. The distribution of test and training trials was same as in Experiment 1.

To preview our findings, the results from Experiments 1 and 2A showed that the visual extrinsic signal could be recruited but not the auditory signal. We wondered whether a more sensitive test would reveal recruitment of the sound cue. To this end we devised an experiment in which the disambiguating stereo information on training trials was kept at threshold levels, to further reduce the reliability of the disambiguating information in training trials [4], [7], [17], [20]. Thus, in Experiment 2B, the duration of the disparity signal was computed for each trial using a three-down-one-up staircase procedure so as to maintain 79% correct performance on training trials. The experiment was conducted as a between-groups design. The 20 participants were randomly assigned to either the learning or the control group (2×N = 10). It was conducted in a single session consisting of six blocks of 80 trials. In the learning group the direction of the pitch glide was perfectly correlated with cube's rotation direction as defined by disparity signals while for the control group the direction of the pitch glide was uncorrelated with its rotation direction. In the learning group the association of rotation direction and pitch glide direction were counterbalanced across participants (5 participants in each subgroup). In order to more precisely control the uncertainty level for each participant, and to guarantee that disambiguating cues were not highly informative, we used a staircase procedure that varied the stereo pulse duration so as to maintain a fixed performance level (79% correct). This dynamic adjustment of the reliability of the stereo cue was different from Experiment 1 and Experiment 2A, in which, the stereo pulse had fixed duration. As a result, participants were not expected to have perfect performance on training trials in Experiment 2B; instead, performance on training trials was controlled by the staircase procedure. It could be argued that this may actually deter any learning since the cube is perceived as rotating in the ‘wrong’ direction 21% of the time. However, in a previous study we have shown that learning can occur with even when the percept is not perfectly correlated with the trained signal [11].

For both groups in Experiment 2B, the stereo-pulse duration was set to 167 ms (20 video frames) at the beginning of the session and was increased or decreased with a step-size of 8.33 ms (1 frame) based on participants' response using a three-down-one-up staircase procedure. The initial duration of the stereo pulse was chosen to ensure that participants would perceive the rotation direction as specified by stereo signals at the beginning of the session. We hypothesized that if the auditory signals were recruited as a cue to rotation direction, then (1) the duration of stereo pulse required to maintain 79% performance level on training trials would be shorter for the group with correlated auditory signals than for the control group, which had uncorrelated auditory signals, and (2) performance on test trials (which were presented only to the right eye and had no stereo) would be correlated with the auditory cue only in the correlated (learning) group. The prediction if the auditory signals are *strongly* recruited is that participants would not require any stereo signals on training trials, while their percepts on the test trials would perfectly determined by the auditory cue. There were 20 test trials interspersed into in the session (See Video S2).

## Results and Discussion

For statistical analyses, each participant's percent perceived-as-cued on test and training trials in Experiment 1 and Experiment 2A was converted to a z-score measure [23], [24]. Appearance probability on ambiguous test trials was computed based on the expected response as predicted by the new signal contingency during training. Saturated performances (0% and 100%) were assigned a Z-score of ±2.326. This Z-score corresponds to 2 responses in 200 trials.

### Experiment 1 – Visual cue-recruitment

**Figure 3:**
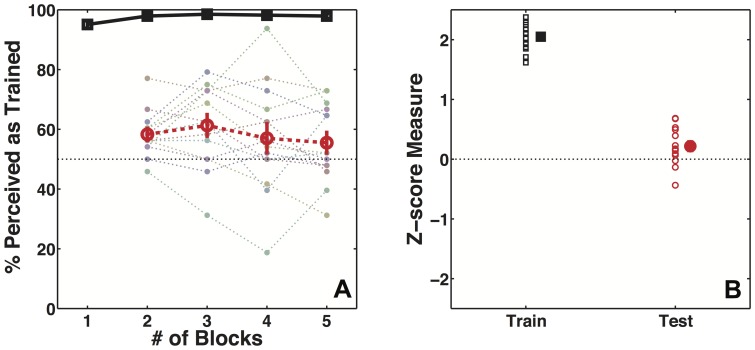
Data show recruitment of extrinsic unimodal signal for construction of visual appearance. A) Participants' (N = 14) mean performance on training (black squares) and test trials (red circles) as function of number of training trials in Experiment 1. The light dotted lines show individual performance for each participant. B) Participants' performance on training (black squares, filled symbol shows mean performance) and test trials (red circles, filled symbol shows mean performance) for the entire session as measured in Z-score units.

In Experiment 1 we used the cue recruitment paradigm to examine whether an extrinsic visual signal can be recruited as a cue to appearance of an ambiguous stimulus, specifically whether the perceived rotation direction of an ambiguous Necker cube can be made contingent on the rotation direction of a surrounding annulus of dots. Figure 3A shows participants' mean performance as a function of number of blocks on disambiguated training and ambiguous test trials. Figure 3B summarizes the performance for the entire session in terms of Z-scores. The short stereo pulse used to disambiguate the stimuli on training trials was effective in controlling participants' percept of the cubes. Participants perceived the stimulus as specified by stereo 97.53% of the time on training trials (z-score  = 2.05, 95% CI [1.91 2.18], t(13) = 32.48, p<<0.0001). Participants maintained fixation well during the experiment, with breaks in fixation occurring only on 3% of the trials on average for all participants.

Participants' percepts on ambiguous test trials were the dependent measure of interest. Percepts were biased in the direction of training contingency, showing that rotation direction of annulus of dots was recruited as a cue to determine rotation direction of the otherwise ambiguous Necker cube (percent of trials  = 58.04%, z-score  = 0.22, 95% CI = [0.04 0.4], t(13) = 2.58, p = 0.023). It has been shown that perceived motion of an ambiguous stimulus can be influenced by motion of neighboring objects [25]–[29]. Therefore, it is possible that a pre-existing bias linking the rotation direction of the annulus of dots to the rotation direction of the cube may have caused the observed effect. If that were true then the strength of learning would have been different for the two groups that were trained with opposite contingency [6], [11]. However, we did not find any difference in participants' performance on training (t(12) = 0.66, p = 0.52) or test trials (t(12) = 1.68, p = 0.12) between the two groups. Further, the effect of learning was in the expected direction for both groups individually, providing further evidence against any preexisting link.

### Experiment 2 – Auditory cue-recruitment

In Experiment 2, we examined whether an extrinsic signal from a different modality (auditory) would be recruited as a cue to visual appearance. Previous attempts to find such an effect have been unsuccessful.

**Figure 4:**
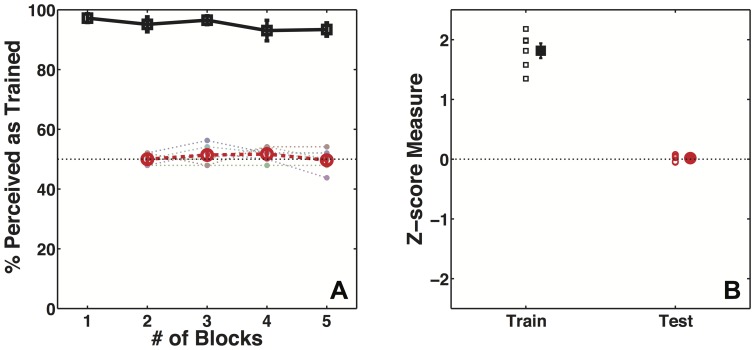
Data show lack of learning for crossmodal extrinsic signal in Experiment 2A. A) Participants' (N = 6) mean performance on training (black squares) and test trials (red circles) as function of number of training trials in Experiment 2A. The light dotted lines show individual performance for each participant. B) Participants' performance on training (black squares, filled symbol shows mean performance) and test trials (red circles, filled symbol shows mean performance) for the entire session as measured in Z-score units.

Figure 4A shows participants' mean performance as a function of number of blocks on disambiguated training and ambiguous test trials. Figure 4B summarizes the performance for the entire session in terms of Z-scores. The short stereo pulse used to disambiguate the stimuli on training trials was effective in controlling participants' percept of the cubes. Participants perceived the stimulus as specified by stereo 95.1% of the time on training trials (z-score  = 1.81, 95% CI [1.5 2.13], t(5) = 14.57, p<<0.0001). However, participants' percept on ambiguous test trials was unaffected by the training contingency (z-score  = 0.02, 95% CI [−0.04 0.07], t(5) = 0.82, p = 0.45). We compared participants' performance on training and test trials in Experiment 1 to that in Experiment 2A. The performance on training trials was very similar between the two groups (t(18) = 1.51, p = 0.15), however the difference in performance on test trials was marginally significant (t(18) = 1.87, p = 0.07). This result shows that while training with low-information stimuli was successful in causing the recruitment of a unimodal extrinsic signal, it failed to cause a crossmodal extrinsic signal to be recruited. Experiment 1 and 2A were identical in all aspects but for the modality of the signal to be recruited and the rotation speed of the cube. It is unlikely that the rotation speed could have caused a difference in the learning outcome, as cue-recruitment has been demonstrated for a wide range of rotation speeds and it has also been shown that rotation speed does not play a critical role in this type of learning [5].

Experiment 2B was conducted as a between-groups experimental design, where we measured the duration of the stereo-pulse required to maintain 79% correct performance for participants in the learning group (auditory signals were perfectly correlated with rotation direction) and for participants in the control group (when auditory signals were uncorrelated with rotation direction).

We excluded data from participants for whom the duration of the stereo pulse was greater than 200 ms at any time during the session, as it implied that we were unable to sufficiently control their percept on training trials. This threshold was sufficient to control appearance in Experiment 1. There were three participants in the control group and one participant in the learning group who failed to meet the criterion.

**Figure 5:**
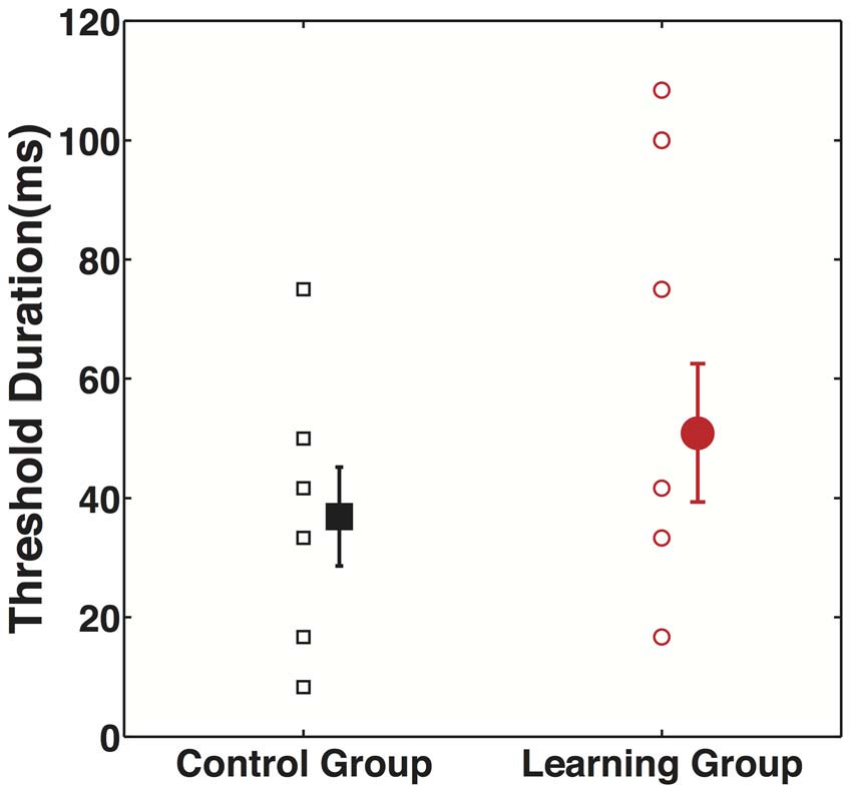
Data show lack of learning for crossmodal extrinsic signal in Experiment 2B. Participants' individual and mean stereo-pulse duration thresholds required maintaining 79% correct performance for Control and Learning groups in Experiment 2B. The data do not agree with our prediction that duration thresholds would be smaller in the learning group, so the sound cue appears not to have been recruited.

Figure 5 shows mean thresholds and individual data for the two groups. The duration thresholds were comparable for both learning and control group (t(14) = 0.8526, p = 0.41), so we did not find evidence that participants learned to use auditory pitch glide direction as a cue to the rotation direction. We also observed no sound-contingent bias on ambiguous test trials (t(8) = 0.31, p = 0.76, mean  = 49.14%, s.e.m = 2.8%).

### General Discussion

The results from Experiment 1 are in stark contrast with our previous study in which no learning of extrinsic cues was observed using similar stimuli and experimental design [3]. The key difference between the two studies is the design of training stimuli. In the current study, the training stimuli were disambiguated using a short stereo pulse, unlike the previous study where stereo signals were present through the entire training trial. In the new study the visual system was forced to resolve the ambiguity using a less reliable stereo signal, and to maintain the percept without the benefit of continued disambiguating information. Previous studies found that low-information stimuli can cause stronger learning [4], [17], [20] and cause learning to occur in cases where it did not occur otherwise [5]–[7]. The result from Experiment 1 confirms the potency of low-information stimuli in promoting the recruitment of difficult-to-learn cues. The current study did not measure the time course of the learning beyond a single session, particularly whether it lasted over night as it does for other cues [2].

Laboratory demonstration of recruitment of a sound cue for visual perception remains an elusive. Experiment 2 constitutes a third failure to find such an effect [2], [3], so we can at this point conclude that the perceptual system is not particularly disposed to learn this particular cross-modal association. However, crossmodal influences do exist, like the McGurk effect [30] or the bounce-pass illusion [31]. Moreover, researchers have found strong evidence for cue-combination across modalities using tasks involving localization [32], [33] and motion perception of multimodal stimuli [33].

In a learning study, Michel and Jacobs [34] showed that judgments of motion direction in a threshold-level random-dot kinematogram task can come to be influenced by auditory signals. However, it is impossible to know where within the system this learning occurred. The sound cues may have influenced only the subjects' final judgments, rather than the visual appearance of the stimulus, because the design did not require the visual system to make a dichotomous decision between alternative perceptual interpretations (i.e. the visual stimuli were not perceptually bistable [23]). It would not be surprising that subjects can learn to use auditory information to answer a question about a visual stimulus when the question is difficult to answer based on visual information alone. Indeed, the authors of [34] conjectured that the auditory cue would be learned under the conditions of their study. They also suggest that the processes underlying the learning observed in their study are distinct from the processes involved in contextual dependent learning such as the one examined in the current study.

The rate and strength of learning for associations between signals and visual appearances lie on a continuum, from fast and readily learned, to unlearnable [18]. Absence of learning for a particular association means that the perceptual system implicitly believes that the signal cannot be informative, or at least should not be used to inform, about the property of the scene represented by the percept. This behavior by a learning mechanism would prevent spurious learning due to coincidental correlations in the environment, at the cost of missing a new reliable cue should one appear. Learning can occur in cases where the additional cues would be useful for disambiguation in the future, as is the case for the low-information training stimuli we used in Experiment 1. In that case the use of low-information training stimuli overcame a reluctance of the system to recruit an extrinsic cue. But this trick does not always work, as was shown by Experiment 2.

## Supporting Information

Video S1 **Example of the visual stimulus used in Experiment 1.**
(MOV)

Video S2 **Example of the visual stimulus used in Experiment 2.**
(MOV)

Audio S1 **Upward pitch glide stimulus used in Experiment 2.**
(WAV)

Audio S2 **Downward pitch glide stimulus used in Experiment 2.**
(WAV)
